# Analyzing Response Times and Other Types of Time-to-Event Data Using Event History Analysis: A Tool for Mental Chronometry and Cognitive Psychophysiology

**DOI:** 10.1177/2041669520978673

**Published:** 2020-12-23

**Authors:** Sven Panis, Filipp Schmidt, Maximilian P. Wolkersdorfer, Thomas Schmidt

**Affiliations:** Experimental Psychology Unit, Faculty of Social Sciences, Technische Universität Kaiserslautern, Kaiserslautern, Germany; Abteilung Allgemeine Psychologie, Fachbereich 06, Psychologie und Sportwissenschaft, Justus-Liebig-Universität Gießen, Giessen, Germany; Experimental Psychology Unit, Faculty of Social Sciences, Technische Universität Kaiserslautern, Kaiserslautern, Germany

**Keywords:** response times, event history analysis, hazard function, conditional accuracy function, speed-accuracy trade-off, survival analysis, transition analysis

## Abstract

In this Methods article, we discuss and illustrate a unifying, principled way to analyze response time data from psychological experiments—and all other types of time-to-event data. We advocate the general application of discrete-time event history analysis (EHA) which is a well-established, intuitive longitudinal approach to statistically describe and model the shape of time-to-event distributions. After discussing the theoretical background behind the so-called hazard function of event occurrence in both continuous and discrete time units, we illustrate how to calculate and interpret the descriptive statistics provided by discrete-time EHA using two example data sets (masked priming, visual search). In case of discrimination data, the hazard analysis of response occurrence can be extended with a microlevel speed-accuracy trade-off analysis. We then discuss different approaches for obtaining inferential statistics. We consider the advantages and disadvantages of a principled use of discrete-time EHA for time-to-event data compared to (a) comparing means with analysis of variance, (b) other distributional methods available in the literature such as delta plots and continuous-time EHA methods, and (c) only fitting parametric distributions or computational models to empirical data. We conclude that statistically controlling for the passage of time during data analysis is *equally* important as experimental control during the design of an experiment, to understand human behavior in our experimental paradigms.

Since the publication of the subtraction method (Donders, 1969) and the additive factors method (Sternberg, 1969), analysis of variance (ANOVA) has become the standard data analysis method in psychology and cognitive (neuro)science for the analysis of response times (RTs). Following these approaches, many researchers interpret differences in RTs between experimental conditions on a difference scale that is assumed to directly capture the time requirements of additional cognitive operations. However, differences in mean RT can only be interpreted that way when assuming that the nature of cognitive processing is captured by the serial information processing framework. Even though the serial information processing framework has been criticized repeatedly in the literature (Cisek & Kalaska, 2010; Eriksen & Schultz, 1979; McClelland, 1979; Pieters, 1983; Schöner et al., 2016), ANOVA continues to be the most popular method to analyze RTs to this day.

As discussed by Van Gelder (1995), there is a viable alternative view on the nature of cognitive processing: Cognition is the behavior of a dynamical system. To understand the behavior of a dynamical system, it is crucial to track its output over time (Schöner et al., 2016). We therefore promote and illustrate the use of a well-established longitudinal or distributional technique known as event history analysis (EHA) for analyzing time-to-event data such as RTs. EHA (also known as survival, hazard, duration, transition, and failure-time analysis) is the name of the standard set of statistical methods for studying *the occurrence and timing of events* in many scientific disciplines (Allison, 2010; Singer & Willett, 2003). While EHA is already applied in many areas of the human sciences, including developmental psychology (Ha et al., 1997), clinical psychology (Corning & Malofeeva, 2004; Greenhouse et al., 1989; Willett & Singer, 1993), social psychology (Griffin, 1993; Keiley & Martin, 2005; Núñez-Antón & Orbe, 2005; Steele et al., 1996, 2004; Stoolmiller & Snyder, 2006), organizational psychology (Morita et al., 1989), and even cognitive psychology (Chechile, 2006; Pannasch et al., 2001; Panis & Wagemans, 2009; Torfs et al., 2010; Wenger & Gibson, 2004; Yang & McConkie, 2001), an introduction to EHA that focuses on its relevance for cognitive (neuro)scientists is still warranted as its use is currently still rather rare. As we will see later, the use of a more advanced and well-established analysis method can maximize the return from the collected data, which is important in view of the costs and time required to run an experiment (Whelan, 2008).

To apply EHA, we must be able to define the event of interest (any qualitative change that can be situated in time), to define time point zero, and to measure the passage of time between time zero and event occurrence in discrete or continuous time units. While sociologists are interested in the occurrence and timing of events such as marriage and divorce—note that some people never marry—and biostatisticians in death, experimental psychologists are interested in events such as button presses (RT analysis), saccade onsets (saccade latency analysis), fixation offsets (fixation duration analysis), and so forth. Typically, time point zero is defined as target display onset time in RT and saccade latency studies. However, sometimes the time of the last response can be defined as time zero for the next response, for example, when studying perceptual dominance durations in studies using ambiguous figures. The onset of fixation is time zero for fixation duration analysis.

The structure of this Methods article is as follows. First, we introduce and explain the concept of hazard, in continuous and discrete time units. Next, we illustrate how to calculate the descriptive statistics in discrete time using a life table, and we discuss two example data sets. We then describe different approaches for obtaining inferential statistics. We end with a discussion of the (dis)advantages of discrete-time EHA, compared with other existing distributional methods.

## The Continuous-Time Hazard Rate Function of Event Occurrence

Luce (1986) mentions that there are several different, but mathematically equivalent, ways to present the information about a continuous random variable T denoting a particular person’s RT in a particular experimental condition, including (a) the cumulative distribution function *F*(t) = P(T ≤ t), (b) its derivative *F*’(t) known as the probability density function *f*(t), (c) the survivor function *S*(t) = 1 – *F*(t) = P(T > t), and (d) the hazard rate function *λ*(t) = *f*(t)/[1 – *F*(t)] = *f*(t)/*S*(t).

In principle, we may present the data as estimates of any of these functions and it should not matter which we use. In practice, it matters a great deal, although that fact does not seem to have been as widely recognized by psychologists as it might be. (Luce, 1986, p. 17)

EHA has been developed to describe and model the hazard function of event occurrence (for RT data, the event is a button-press response). For continuous RT data, hazard quantifies the instantaneous risk that a response will occur at time point t, given that it has not occurred before time t. In other words, it quantifies the likelihood that a response we are still waiting for at time t will occur within the next instant. Just as speed is defined as a rate—the distance covered per unit time—so too is the continuous-time hazard *λ*(t). For example, if time is measured in seconds and *λ*(3.0) = 2, then the instantaneous rate of event occurrence equals two events per second after 3 seconds of waiting time. There are at least five reasons why statisticians and mathematical psychologists recommend focusing on the hazard function in practice, when dealing with a finite sample of time-to-event data.

**Figure 1.**
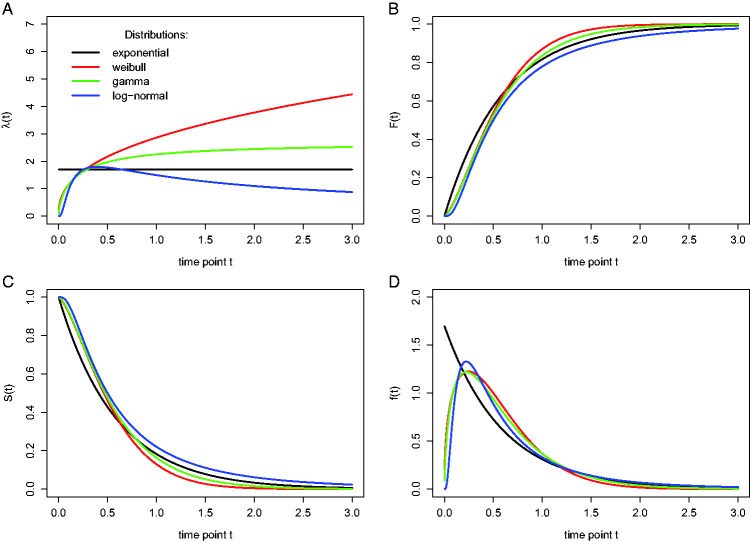
Four views on four different waiting-time distributions in continuous time. The hazard rate function *λ*(t) (A), the cumulative distribution function *F*(t) (B), the survivor function *S*(t) (C), and the probability density function *f*(t) (D) are shown for each of four theoretical probability distributions (different colors: exponential, Weibull, gamma, log-normal). While the hazard rate function for the exponential is flat, it keeps increasing for the Weibull, it increases to an asymptote for the gamma, and it reaches a peak and then gradually decreases to an asymptote for the log-normal.

First, the hazard function of response occurrence is one of the most diagnostic functions when describing the distribution of a sample of time-to-event data (Allison, 2010; Luce, 1986; Townsend, 1990). For example, “the hazard function itself is one of the most revealing plots because it displays what is going on locally without favoring either short or long times, and it can be strikingly different for *f*’s that seem little different” (Luce, 1986, p. 19). To illustrate this, Figure 1 shows the *F*(t), *f*(t), *S*(t), and *λ*(t) for four theoretical waiting-time distributions. In contrast to *λ*(t), all *F*(t) and *S*(t) distributions look vaguely alike, and we cannot easily describe salient features other than the mean and standard deviation. Also, the density function *f*(t) conceals what is happening in the right tail of the distribution (Luce, 1986). As discussed by Holden et al. (2009), “probability density functions can appear nearly identical, both statistically and to the naked eye, and yet are clearly different on the basis of their hazard functions (but not vice versa). Hazard functions are thus more diagnostic than density functions” (p. 331).

Second, because RT distributions may differ from one another in multiple ways, Townsend (1990) developed a dominance hierarchy of statistical differences between two arbitrary distributions A and B. For example, if *F*_A_(t) > *F*_B_(t) for all t, then both cumulative distribution functions are said to show a complete ordering. Townsend (1990) showed that a complete ordering on the hazard functions—*λ*_A_(t) > *λ*_B_(t) for all t—implies a complete ordering on both the cumulative distribution and survivor functions—*F*_A_(t) > *F*_B_(t) and *S*_A_(t) < *S*_B_(t)—which in turn implies an ordering on the mean latencies—mean A < mean B. In contrast, an ordering on two means does *not* imply a complete ordering on the corresponding *F*(t) and *S*(t) functions, and a complete ordering on these latter functions does *not* imply a complete ordering on the corresponding hazard functions. This means that stronger conclusions can be drawn from data when comparing the RT hazard functions using EHA. For example, when mean A < mean B, the hazard functions might show a complete ordering (i.e., for all t), a partial ordering (e.g., only for t > 300 ms, or only for t < 500 ms), or they may cross each other one or more times.

Third, EHA does not discard right-censored observations when estimating hazard functions, that is, trials for which we do not observe a response during the data collection period so that we only know that the RT must be larger than some value. This is important because although a few right-censored observations are inevitable in most RT tasks, a lot of right-censored observations are expected in experiments on masking, the attentional blink, and so forth, for example.

There are other types of censoring. Left censoring occurs when all that is known about an observation on a variable T is that it is *less* than some value. Interval censoring combines right and left censoring so that all you know about T is that a < T < b, for some values of a and b (Allison, 2010). Random censoring occurs when observations are terminated for reasons that are not under the control of the experimenter.

Importantly, all standard statistical methods for time-to-event data require that random censoring be noninformative: For example, a trial that is censored at time c should be representative of all those trials with the same values of the explanatory variables that survive to c (Allison, 2010). For example, the occurrence of an equipment error during a trial will introduce random censoring that is uninformative. However, when estimating the hazard of correct response occurrence, error responses introduce random censoring (and vice versa) that is very likely informative, because response channels are known to compete with one another (Burle et al., 2004; Eriksen et al., 1985; Praamstra & Seiss, 2005). We therefore never recommend to describe or model the hazard of correct response occurrence independently from the hazard of error response occurrence but to extend the hazard of response occurrence with conditional accuracy functions (see later).

The most common type of right-censoring is “singly Type I censoring” that applies when the experiment uses a fixed response deadline for all trials. “Type I” means that the censoring time is fixed and is under the control of the experimenter, and “singly” refers to the fact that all observations have the same censoring time (Allison, 2010). Discarding such trials—or trials with very long RTs in case the experimenter waits for a response on each trial—may introduce a sampling bias that results in underestimation of the mean. In contrast, EHA can include the data information from all trials when estimating the descriptive statistics.

Fourth, hazard modeling allows incorporating *time-varying* explanatory covariates such as heart rate, electroencephalogram (EEG) signal amplitude, gaze location, and so forth (Allison, 2010) which is useful for cognitive psychophysiology (Meyer et al., 1988). For more information, see Singer and Willett (2003, pp. 426–442) and Allison (2010, pp. 243–246).

Finally, hazard is more suited as a measure of the concept of processing capacity, that is, the amount of work the observer is capable of performing within some unit of time (Wenger & Gibson, 2004). The hazard function can capture the notion of the instantaneous capacity of the observer for completing the task in the next instant, given that the observer has not yet completed the task.

## The Discrete-Time Hazard Probability Function of Event Occurrence

Unfortunately, estimating the shape of the continuous-time hazard rate function for one observer in one experimental condition is not straightforward because one needs at least 1,000 trials for example (Bloxom, 1984; Luce, 1986; Van Zandt, 2000). However, by shifting to discrete time, we can trade-off some temporal resolution for increased applicability of EHA in RT studies that typically collect less than 1,000 trials per condition per participant. In this Methods article, we therefore focus on the application of *discrete-time hazard analysis* to RT data, which is straightforward, easy, and intuitive and allows for flexible statistical modeling by logistic regression which is highly familiar to psychologists (Allison, 1982, 2010; Singer & Willett, 1991, 2003; Willett & Singer, 1993, 1995).

**Figure 2.**
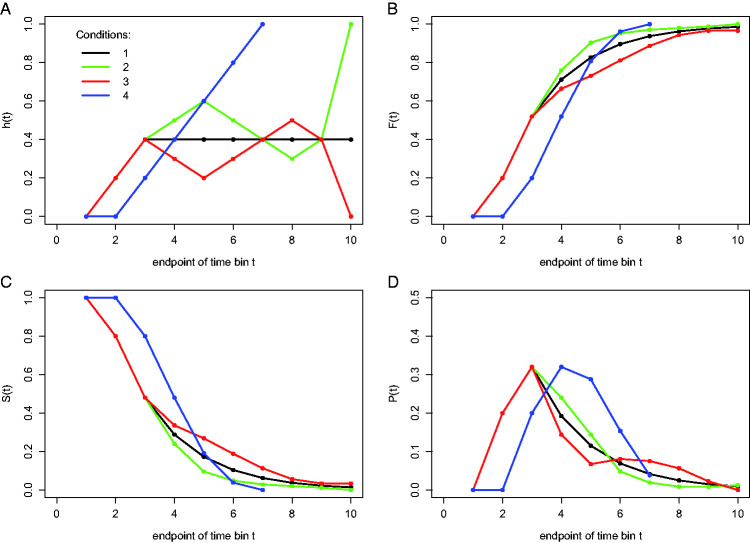
Four views on four different waiting-time distributions in discrete time. The hazard probability function *h*(t) (A), the cumulative distribution function *F*(t) (B), the survivor function *S*(t) (C), and the (sub)probability mass function *P*(t) (D) are shown for each of four hypothetical conditions (different colors).

In Figure 2A, four hypothetical discrete-time population hazard functions are plotted with time divided in 10 discrete bins (1–10). Each function was constructed by selecting a series of 10 real numbers from the interval [0,1] with replacement, with the only restriction that once “1.0” is selected then the following numbers are set to “missing data”—the reader can construct her or his own example functions. Each hazard function completely describes the shape of a distribution of discrete waiting times. For example, the four theoretical functions in Figure 2A could reflect the true RT distributions of a single participant in four experimental conditions (studied with a small-*N* design; in which a large number of observations are made on a relatively small number of experimental participants, Smith & Little, 2018); in this example, time might have been measured in discrete time bins of 50 ms each, with a censoring time of 500 ms. Or they might reflect the true distributions of the time it takes to earn a first doctoral degree measured in years for four groups of 100 participants with certain personality characteristics (large-*N* design), with a censoring time of 10 years. In each case, *h*(t) gives the conditional probability that the event of interest occurs in bin t given that it has not yet occurred before, or *h*(t) = P(T = t|T ≥ t), where T is a discrete random variable denoting the rank of the time bin in which the event occurs. The discrete-time hazard function of event occurrence thus tells us the probability that the event we are still waiting for (at the start of bin t) will actually occur in bin t.

Figure 2C displays the corresponding discrete-time survivor functions, or *S*(t) = P(T > t) = [1 – *h*(t)] * [1 – *h*(t–1)] * … * [1 – *h*(1)], which gives for each bin the probability that the response does not occur before the end of bin t. The survivor function is the complement of the cumulative distribution function (Figure 2B), or *S*(t) = 1 – *F*(t) = 1 – P(T ≤ t). Figure 2D shows the corresponding probability mass functions, or *P*(t) = P(T = t) = *h*(t) * *S*(t−1).

We constructed the hazard functions in Figure 2A in such a way that they show some symmetry. For example, Condition 1 (black line) might represent a neutral priming condition and Conditions 2 and 3 a congruent and incongruent priming condition, respectively. Let us assume for simplicity that each bin is 1 second wide and that the censoring time equals 10 seconds so that we have the following sequence of bins: (0,1], (1,2], … , (9,10]. For example, the discrete-time hazard for bin 2 in the neutral condition equals .20 (for bins 1–3, the hazard functions for the first three conditions lie on top of each other). In other words, given that time has passed until 1 second after target onset without response occurrence, then there is a conditional probability of .2 that the response occurs sometime during the next second, that is, in the second bin or time segment (1,2]. In short, *h*(2) = .2. When the waiting time has increased to 2 seconds, *h*(3) = .4, and so forth.

If we now compare Conditions 2 and 3 (green and red lines), we see a large positive priming effect in hazard for time segment (3,6] followed by a smaller negative (i.e., inverted) priming effect for time segment (7,8]. Note that while the hazard functions for Conditions 2 and 3 cross two times, the *S*(t) and *F*(t) functions do not cross, because they cumulate the (complement of the) current and previous hazard values. This implies that also quantile plots and delta plots—and other types of visualization based on plotting and comparing quantiles from *F*(t)—would not be able to reveal the crossing that is visible in hazard.

Similarly, note that the symmetry present in the hazard functions for the first three conditions is also absent in the *P*(t) functions. As a matter of fact, if we would only study *P*(t), we might conclude incorrectly that the late negative priming effect lasts longer than the early positive priming effect. However, the *P*(t) values do not give any information on the time course of event occurrence because they denote the probability that the event occurs in bin t given that it can occur in *any* (previous, current, or future) bin. In other words, they simply tell you how many percent of all trials will experience the event in bin t. Note that the *P*(t) values in Figure 2D do not sum to 1 for Conditions 1 and 3, which is why these are called *subprobability* mass functions (Chechile, 2003); also, the corresponding *h*(t) and *F*(t) functions do not reach 1, and the *S*(t) functions do not reach zero.

## Obtaining Descriptive Statistics for Discrete Time Units: The Life Table

To calculate the descriptive statistics—functions of discrete time—for a finite time-to-event data set, one has to set up a *life table*. In the context of a small-*N* design, the life table summarizes the history of event occurrences for a combination of participant and experimental condition. To set up a life table, you need to determine the censoring time and divide it up into a sequence of contiguous time bins. The fixed censoring time point is typically the response deadline used, or a time point after which you expect no useful responses anymore in any trial of any condition. In this section, we shortly discuss real data from two published experiments using a small-*N* design, one on masked priming, and one on visual search.

### Masked Priming

**Figure 3.**
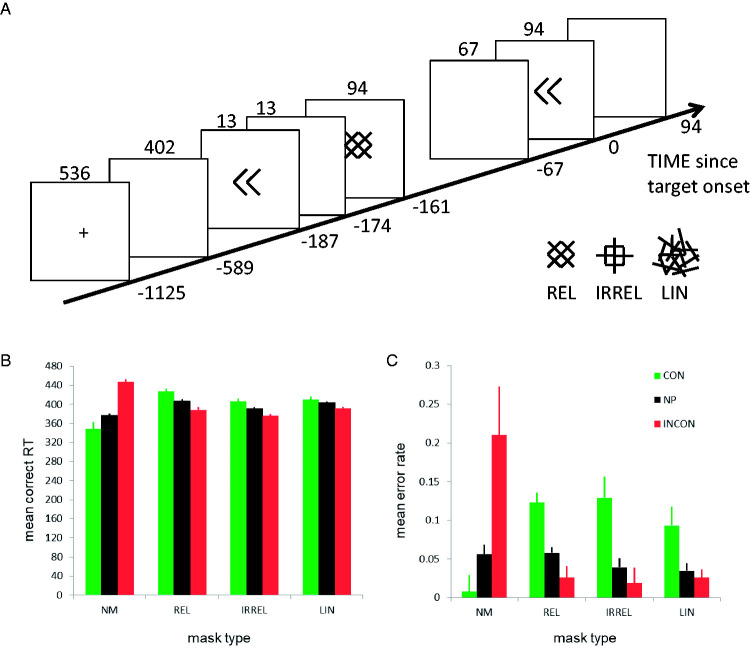
Masked priming example. (A) Trial and mask designs used in Experiment 1 of Panis and Schmidt (2016). A trial with a congruent prime and a relevant mask is shown. Insets show three mask types. Time on the *x* axis is measured in milliseconds relative to target onset. (B) Mean correct RT. (C) Mean error rate. Error bars represent ± 1 SEM corrected for between-subject variation. NP = no prime; CON = congruent prime; INCON = incongruent prime; NM = no mask; REL = relevant mask; IRREL = irrelevant mask; LIN = random lines mask.

Panis and Schmidt (2016) asked participants to perform speeded keypress responses to the direction of a 94 ms double arrow target (left/right), within 600 ms (Figure 3A). The central target could be preceded by a central 13 ms double arrow prime that was followed by a 94-ms pattern mask. The factors prime type and mask type were manipulated factorially. The prime could point in the same direction as the target (CONgruent), in the opposite direction (INCONgruent), or no prime was presented (NP). The mask stimulus could be response-relevant (REL), response-irrelevant (IRREL), a set of random lines (LIN), or no mask was presented (NM). Consistent with the literature, the mean correct RT (Figure 3B) and mean error rates (Figure 3C) show a positive priming or congruency effect (PCE) of about 100 ms and 20 percentage points when no mask was presented, but the reversed effect in the presence of relevant or irrelevant masks: a negative congruency effect (NCE) of about –40 ms and –10 percentage points.

Table 1 presents the life table for the data of a single participant in condition NP-NM (no prime, no mask). The first 600 ms after target onset are divided into 15 bins of 40 ms indexed by t = 1 to 15. After counting the number of responses in each bin, one can then directly estimate the discrete-time hazard probability function of response occurrence: *h*(t) = P(T = t | T ≥ t), where T ≥ t denotes the event that the response does not occur before the start of bin t. For each bin t, the sample-based estimate of *h*(t) is obtained by dividing the number of observed responses in bin t by the *risk set* of bin t, which is the number of trials that are still response-free at the start of bin t. Note that the four right-censored observations—trials without response occurrence for which we only know that RT must be larger than 600 ms—do contribute to the risk set of each bin (ignoring such trials creates a sampling bias). Also note how the standard error of *h*(t) tends to increase as the waiting time increases, because the risk set is becoming rather small for later time bins.

**Table 1.** Example Life Table for Discrete-Time Statistics.

bin	t	rc	E	RS	h(t)	se[h(t)]	*S*(t)	se[S(t)]	P(t)	se[P(t)]	# correct	# error	ca(t)	se[ca(t)]
(0,40]	1	0	0	220	0	0	1	0	0	0	0	0	NA	NA
(40,80]	2	0	0	220	0	0	1	0	0	0	0	0	NA	NA
(80,120]	3	0	0	220	0	0	1	0	0	0	0	0	NA	NA
(120,160]	4	0	0	220	0	0	1	0	0	0	0	0	NA	NA
(160,200]	5	0	0	220	0	0	1	0	0	0	0	0	NA	NA
(200,240]	6	0	0	220	0	0	1	0	0	0	0	0	NA	NA
(240,280]	7	0	7	220	0.032	0.012	0.968	0.012	0.032	0.012	2	5	0.29	0.171
(280,320]	8	0	13	213	0.061	0.016	0.909	0.019	0.059	0.016	10	3	0.77	0.117
(320,360]	9	0	26	200	0.130	0.024	0.791	0.027	0.118	0.022	24	2	0.92	0.052
(360,400]	10	0	40	174	0.230	0.032	0.609	0.030	0.182	0.026	40	0	1	0
(400,440]	11	0	48	134	0.358	0.041	0.391	0.028	0.218	0.028	47	1	0.98	0.021
(440,480]	12	0	37	86	0.430	0.053	0.223	0.020	0.168	0.025	37	0	1	0
(480,520]	13	0	32	49	0.653	0.068	0.077	0.010	0.145	0.024	32	0	1	0
(520,560]	14	0	9	17	0.529	0.121	0.036	0.005	0.041	0.013	9	0	1	0
(560,600]	15	4	4	8	0.500	0.177	0.018	0.003	0.018	0.009	4	0	1	0

*Note*. This life table is based on the 220 trials of Participant 6 in the target-only condition (NP-NM) of Experiment 1 of Panis and Schmidt (2016). For each time bin (column 1) with rank t (column 2), the number of observed responses (E) are counted, and the risk set (RS) is determined, before estimating (a) the discrete-time hazard function *h*(t) = P(T = t|T ≥ t) as E/RS, (b) the survivor function *S*(t) = P(T > t) = [1-*h*(t)] * [1-*h*(t-1)] * … * [1-*h*(1)], and (c) the probability mass function *P*(t) = P(T = t) = *h*(t)*S(t-1). The standard errors for *h*(t), *P*(t), and *ca*(t) are estimated using the familiar formula for a proportion p—the square root of {p(1-p)/N}—where N equals RS(t) for *h*(t), RS(1) for *P*(t), and E(t) for *ca*(t). The standard errors for *S*(t) are estimated using the recurrent formula on page 350 of Singer and Willett (2003). Note that *P*(t) also equals the number of events in bin t divided by the risk set of the first bin: *P*(t) = E(t)/RS(1). Four trials are right-censored (rc) at 600 ms after target onset (column 3), that is, no response occurred for these trials during the 600 ms data collection period so that we only know that RT > 600 ms. At time point zero, *S*(0) = 1, *P*(0) = 0, and *h*(0) is undefined (NA). It is important to realize the difference between the probability mass and the hazard function. The (sub)probability mass function calculates the response count in a given time bin relative to the number of all trials, whereas the hazard function calculates the response count relative to the number of all trials that are still response-free up to the start of that time bin.

Because we are dealing with two-button discrimination data, the *h*(t) analysis of response occurrence is extended with an analysis of conditional accuracy, that is, the microlevel speed-accuracy trade-off function (Allison, 2010; Pachella, 1974; Wickelgren, 1977). The conditional accuracy function, or *ca*(t) = P(correct|T = t), is the conditional probability that an observed response is correct given that it occurs in bin t and is estimated by dividing the number of correct responses in bin t by the number of observed responses in bin t (Table 1). By using *h*(t) functions in combination with *ca*(t) functions, one can provide an unbiased, time-varying, and probabilistic description of the latency and accuracy of *any* sample of (right-censored) event times.

**Figure 4.**
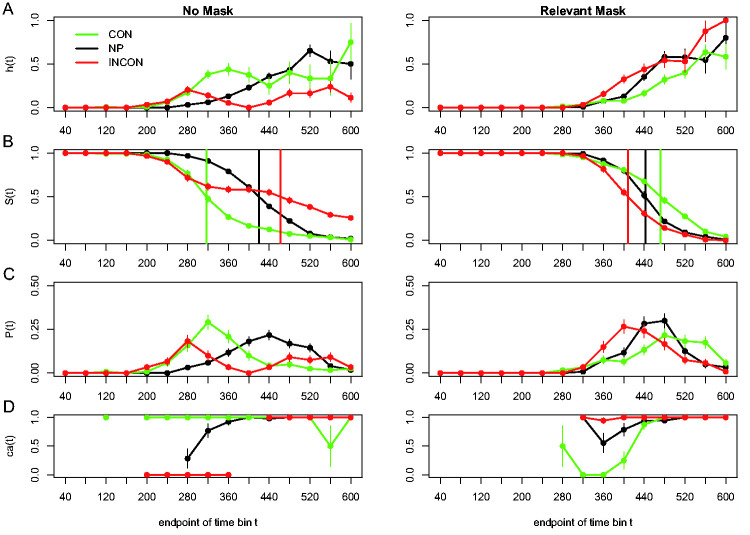
Sample-based estimates for Participant 6 in Experiment 1 of Panis and Schmidt (2016). For each combination of mask type (no mask and relevant mask) and prime type (congruent, no prime, incongruent), the estimated discrete-time hazard function *h*(t) is plotted (A), together with the survivor function *S*(t) (B), the (sub)probability mass function *P*(t) (C), and the conditional accuracy function *ca*(t) (D). Time axes are relative to target onset. Error bars represent ± 1 standard error of the respective proportion. CON = congruent prime; NP = no prime; INCON = incongruent prime.

Sample-based estimates of *h*(t), *S*(t), *P*(t), and *ca*(t) are shown for one participant in Figure 4, for two mask conditions (none and relevant) and three prime types (No Prime, CONgruent, INCONgruent). We refer to each bin using its endpoint, for example, the hazard estimate for bin (240,280] is *h*(280). Figure 4 offers a fascinating view into the microgenesis of primed responses. In the no mask conditions (left panels), response onset is much earlier when primes are present, and the upswing in response hazards is at first identical for consistent and inconsistent primes. If such an early response is emitted, it is always correct for congruent primes and always incorrect for incongruent primes, as shown by the *ca*(t) functions. This clearly indicates that these initial responses are triggered exclusively by the prime without any contribution from the target (the crucial prediction of the rapid-chase theory of response priming; Schmidt et al., 2006, 2011, 2015).

Once the waiting time has reached 280 ms after target onset without response occurrence, response hazards continue to increase temporarily for congruent primes but start to decline for incongruent primes and eventually even reach zero: in bin (360,400] after target onset, no responses are emitted when the prime is incongruent. In our view, this temporary decline in hazard reflects—at least initially—response competition from the target, which is becoming overtly available in bin 280 and activates the opposite (correct) response as the prime. In other words, this is the phase where the target starts taking over response control from the prime. After bin 400, *h*(t) starts to increase again in the incongruent condition, and if such a late response is emitted, it is always correct. Thus, the response conflict has been resolved in favor of the target, and these late responses are controlled entirely by the target’s identity.

But something else is going on in the relevant mask condition (right panels). The first overt responses only appear around 320 ms after target onset. Overall, response hazards increase faster in incongruent than in congruent trials (with the no-prime condition in between), demonstrating a reversed priming or NCE in response occurrence. Moreover, the earliest emitted responses are typically correct in incongruent trials and typically incorrect in congruent trials: a complete reversal of the pattern in the no mask condition. When the target information becomes available, it now delays responses in the congruent condition around 360 ms after target onset. Following this temporary dip, *h*(t) sharply increases, and all responses emitted after 480 ms are correct.

**Figure 5. fig5-2041669520978673:**
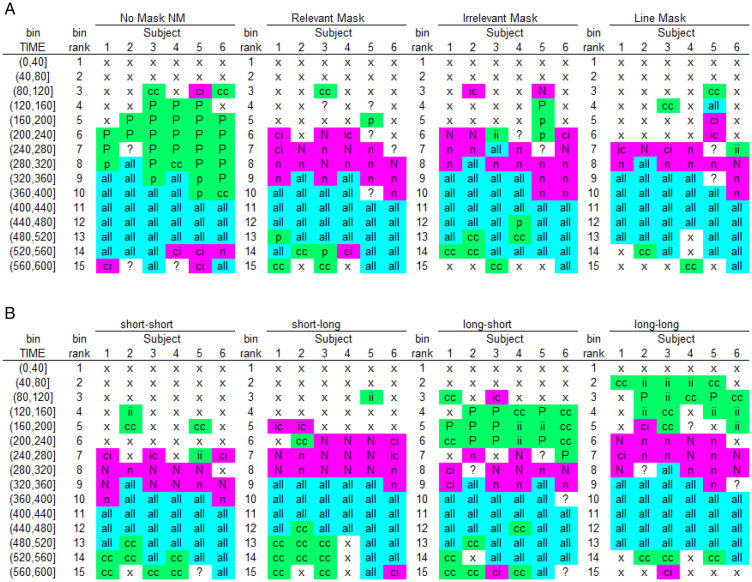
Sample-based *ca*(t)-state transition plots. For each participant, bin, and mask type (A, Experiment 1) or SOA combination (B, Experiment 2), we first coded the type of difference in observed performance in *ca*(t) between congruent (CON) and incongruent (INCON) prime conditions and then applied a color code (green = evidence for PCE; pink = evidence for NCE; cyan = no evidence for either). Specifically, for bins where responses are observed for both CON and INCON: “P”: *ca*(t) = 1 for CON and *ca*(t) = 0 for INCON; “p”: CON minus INCON ≥ .2; “N”: *ca*(t) = 0 for CON and *ca*(t) = 1 for INCON; “n”: CON minus INCON ≤ –.2; “all”: *ca*(t) > .8 for both CON and INCON. For bins where responses exclusively occur in either CON or INCON: “cc”: *ca*(t) = 1 for CON and no responses for INCON; “ii”: no responses for CON and *ca*(t) = 0 for INCON; “ic”: *ca*(t) = 0 for CON and no responses for INCON; “ci”: no responses for CON and *ca*(t) = 1 for INCON. Remaining bins: “x”: no responses observed in CON and INCON; “?”: other cases. The reader can compare the codes for Participant 6 in Figure 5A (relevant and no mask) with Figure 4D. NM = no mask.

The hazard functions for congruent and incongruent trials thus show a partial ordering (i.e., only for t > 280 ms in the no mask condition, and for t > 320 ms in the relevant mask condition). In other words, the hazard functions reveal the onset time, duration, and shape of the behavioral effect. The differences in means also typically underestimate the duration of the effect in terms of hazard. For example, the within-trial duration of the PCE when the mask is absent is at least 200 ms (5 bins) and that of the NCE when the mask is relevant is at least 160 ms (4 bins). Also, plotting hazard and conditional accuracy functions can reveal important interindividual differences and the time-locking of effects to stimuli or other events. For example, Panis and Schmidt (2016) compared the dynamics of the priming effect in the *ca*(t) functions for the six different participants and found a high similarity (Figure 5A): Every participant showed a temporary PCE in the no mask condition and a temporary NCE in the various masking conditions. Figure 5B shows the result of a second experiment where the prime-mask and mask-target stimulus-onset-asynchronies (SOAs) were varied independently. The plot shows that three distinct states can be identified when the prime-mask SOA is long (conditions “long–short” and “long–long”): a PCE state time-locked to prime-onset, an NCE state time-locked to mask onset, and an “all correct” state time-locked to target onset. Note that the same three states have been observed in the Lateralized Readiness Potential by Jaśkowski et al. (2008) and Eimer and Schlaghecken (1998). Crucially, the NCE appears ∼360 ms after mask onset in every condition, an estimate very similar to the 350 ms estimate obtained by looking at pointing movement trajectories (Schmidt et al., 2015).

Panis and Schmidt (2016) concluded that the NCE is neither caused by automatic self-inhibition of the primed response due to backward masking nor by updating response-relevant features of the mask, but by active, selective mask-triggered inhibition. The mask thus acts as a *stop-signal* within the current task context that initiates selective inhibition of the premature prime-triggered response, which temporarily disinhibits the opposite response (thrust reversal; Schmidt et al., 2015). Importantly, these distributional results are compatible with a computational model of the basal ganglia, a subcortical collection of nuclei that are involved in response gating and (selective and global) response inhibition (Wiecki & Frank, 2013).

### Visual Search

**Figure 6.**
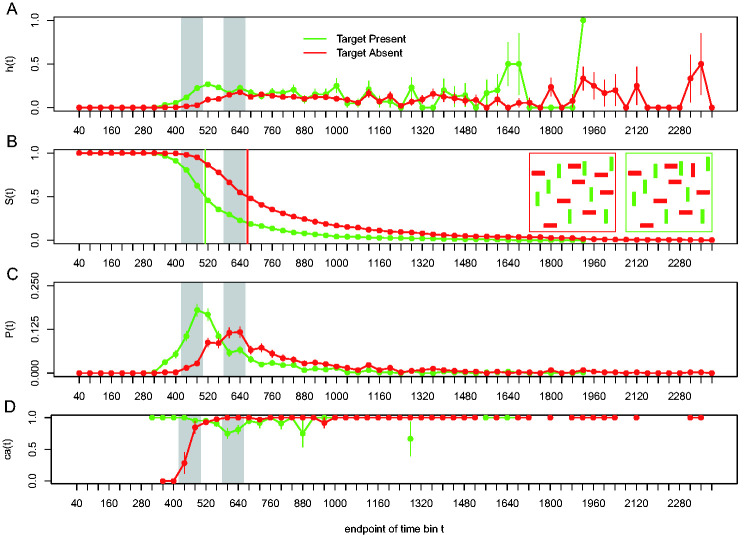
Visual search example. The data for one representative participant in each of the target-present and target-absent conditions of the color-orientation conjunction search task with set size 18 of Wolfe et al. (2010) are plotted as (A) hazard function *h*(t), (B) survivor function *S*(t), (C) (sub)probability mass function *P*(t), and (D) conditional accuracy function *ca*(t). Both insets in Figure 6B show example displays. The target is a vertical red object. The passage of time is measured discretely using bins of 40 ms starting at search display onset. The vertical lines in Figure 6B show the estimated median response times for the target-present and target-absent conditions. The gray surface areas are used for interpretation (see main text). Error bars represent ± 1 standard error of the respective proportion.

Panis et al. (2020) reanalyzed the benchmark visual search data sets collected by Wolfe et al. (2010). For example, in the color-orientation conjunction search task, 10 participants searched a single display for a red vertical rectangle among green vertical and red horizontal rectangles. Four different set sizes (target plus distractors; 3, 6, 12, or 18) were randomly intermixed. Participants pressed one key if the target was present (50% of trials) and another if the target was absent. They were instructed to respond as quickly and correctly as possible and received feedback after each trial. Accuracy and RT in ms were recorded. Each participant provided approximately 10 blocks of 400 trials, leading to about 500 trials per participant and search condition. Figure 6 shows the data for one representative participant in the color-orientation conjunction search task with a set size of 18 objects, using bins of 40 ms and a censoring time of 2,400 ms.

First, there is only a partial ordering of the hazard functions with respect to the effect of target presence (only for t < 600 ms), and the hazard functions are relatively flat for the right tail of the RT distributions. Second, false alarms occur mostly early in time, while misses occur mostly for medium-latency responses. The miss rate peaks around 600 ms after search display onset. As far as we know, none of these features of visual search behavior are predicted by current cognitive models of visual search (Panis et al., 2020).

One tentative interpretation of these data is based on the idea that behavior at any point in time is determined not only by the outcome of the ongoing search process but also by response biases and reactive cognitive control processes (Panis et al., 2020). For example, we can distinguish five phases in the time-dispersed behavior of this observer (the gray surface areas in Figure 6 mark phases two and four). First, if the waiting time has increased until 360 ms after search display onset, then *h*(400) is higher for target-present than target-absent trials, and all emitted responses are correct for target-present, but incorrect for target-absent trials. The earliest responses thus show a strong yes-bias, regardless of target presence. Kiss et al. (2012) concluded that the attentional selection of targets that are defined by a combination of features—here: “red” and “vertical”—is a two-stage process: Attention is initially captured by all target-matching features but is then rapidly withdrawn from distractor objects that share some but not all features with the current target. This suggests that at the end of the initial feedforward sweep of processing right after display onset, all elements in the search display will have captured attention to some extent, each signaling the presence of target features such as red and/or vertical in the conjunction search task. This explains the presence of the early yes-response bias. We also assume that the target is indeed found on a few of the target-present trials (e.g., those where the target is very salient due to spatial grouping processes), which explains the higher hazard for target-present trials. If no early response occurs, however, time passes on, and the search continues.

Second, in the time range 400–480 ms, hazard further increases for both conditions, while *ca*(t) quickly increases above chance level for target-absent trials and starts to slightly decrease for target-present trials. Thus, while the search process might finish on a subset of trials in this time range, Panis et al. (2020) suggested that online error-monitoring processes can detect the task-interfering yes-response bias in the earliest response tendencies and that reactive cognitive control processes such as active and selective response suppression kick in (Panis & Schmidt, 2016). The active and selective suppression of the premature yes-response tendency can result in a temporary disinhibition of the competing no-response, which would lead to an overt no-response if a momentary threshold is crossed on some trials. Thus, in those trials where the search process has not yet finished, this suppression can lead to overt misses in target-present trials, and it can explain the sharp increase in *ca*(t) for target-absent trials, which is presumably too early to reflect pure correct rejection decisions.

Third, in the time range 480–560 ms, performance is optimal in the sense that (a) hazard is at its highest level so far, and (b) conditional accuracy is high for both target presence conditions. Around this point in time after display onset, behavior is thus determined mostly by the outcome of the search process. However, for a subset of trials, no overt decision is made and time passes on.

Fourth, in the time range 560–640 ms, the difference in hazard disappears, and a no-bias develops as the miss rate reaches a maximum, and there are no false alarms. In other words, if the waiting time has increased until 560 ms, then *h*_TP_(600) equals *h*_TA_(600), and *ca*_TP_(600) = .8 while *ca*_TA_(600) = 1. Thus, for the more difficult search trials, the suppression effects accumulate—causing hazard to decrease and the miss rate to peak in the target-present condition, while more and more correct rejection decisions occur when the target is absent.

Finally, after 640 ms, hazard functions are flat and most emitted responses are correct. In other words, the system quits the search and finally transitions to a state with flat hazard functions without a systematic effect of target presence. Horizontally shaped hazard functions point to exponentially distributed RTs. Based on the findings of Shenoy et al. (2013), we assume that these flat right tails reflect RT outliers during decision making. Shenoy et al. (2013) described neuronal motor activity in macaque monkeys from a dynamical systems perspective by studying single-trial neural trajectories in a state space. They found that the neural state wanders before falling back on track in RT outlier trials so that the monkey hesitated for an abnormally long time before movement onset. Interestingly, Thompson et al. (1996) found that much of the RT variance in search tasks is due to postperceptual motor processing, perhaps to provide the adaptive advantage of allowing for subsequent visual processing and cognitive factors to alter the response choice before an irrevocable commitment is made. For example, one might keep inspecting a few more items even though the no-response is already selected in the target-absent condition. Similarly, one might explicitly compare the presumed target with a few surrounding distractors to confirm target presence, even though the yes-response is already selected in the target-present condition.

Both these and other discrete-time EHA studies of simultaneous masking (Panis & Hermens, 2014), object recognition (Panis et al., 2017; Panis & Wagemans, 2009; Torfs et al., 2010), spatial cueing (Panis, 2020; Panis & Schmidt, 2020), and priming (Wolkersdorfer et al., 2020) teach us three things: (a) Mean performance measures conceal crucial information about behavioral dynamics such as premature response activation, time-locking, response suppression, and how performance changes as time passes by within *and* across trials, (b) RT and accuracy data reflect different aspects of the time-dispersed decision process (Mulder & van Maanen, 2013), and (c) sometimes one can identify subsets of participants that display qualitatively different behavior (Miller & Schwarz, 2018; Panis, 2020; Panis et al., 2020).

Note that when you measure time in continuous units, the survivor function *S*(t) can be estimated nonparametrically using the Kaplan–Meier method (Kaplan & Meier, 1958). Estimates of the hazard *rate* function can be obtained based on Kaplan–Meier but are typically smoothed to some extent because they tend to be very choppy when not based on sufficient data (Allison, 2010).

## Obtaining Inferential Statistics

There are several approaches for obtaining inferential statistics (Allison, 2010; Austin, 2017). When you simply want to compare survival functions between two groups in continuous time (large-*N* design), the log-rank and the Wilcoxon tests are available (the latter puts more weight on earlier points in time).

When you want to study how hazard depends on various predictors, you can fit regression models to the data (Singer & Willett, 2003). An example *discrete-time* hazard model with three predictors (TIME, X_1_, X_2_) and the complementary log-log (cloglog) link function can be written as follows:

$$\begin{array}{l} \text{cloglog}\left[h\left(\text{t}\right)\right]=\text{ln}\left(-\text{ln}\left[\text{1}-h\left(\text{t}\right)\right]\right)=\;[\alpha_{0}\text{ONE}+\alpha_{\text{1}}(\text{TIME}-\text{1})\\ \;+\alpha_{\text{2}}(\text{TIME}-\text{1}{)}^{\text{2}}+\alpha_{\text{3}}(\text{TIME}-\text{1}{)}^{\text{3}}]+[\beta_{\text{1}}{\text{X}}_{\text{1}}+\beta_{\text{2}}{\text{X}}_{\text{2}}+\beta_{\text{3}}{\text{X}}_{\text{2}}(\text{TIME}-\text{1})]. \end{array}$$

The main predictor variable TIME is the time bin index t (see Table 1) that is centered on value 1 in this example. The complementary log-log link is preferred over the logit link when events can occur in principle at any time point within a bin, which is the case for RT data (Singer & Willett, 2003). The first set of terms within brackets, the alpha parameters multiplied by their polynomial specifications of (centered) time, represents the shape of the baseline cloglog-hazard function (i.e., when all predictors X_i_ take on a value of zero). The second set of terms (the beta parameters) represents the vertical shift in the baseline cloglog-hazard for a 1 unit increase in the respective predictor. Predictors can be discrete, continuous, and time-varying or time-invariant. For example, the effect of a 1 unit increase in X_1_ is to vertically shift the whole baseline cloglog-hazard function by β_1_ cloglog-hazard units. However, if the predictor interacts linearly with time (see X_2_ in the example), then the effect of a 1 unit increase in X_2_ is to vertically shift the predicted cloglog-hazard in bin 1 by β_2_ cloglog-hazard units (when TIME–1 = 0), in bin 2 by β_2_+ β_3_ cloglog-hazard units (when TIME–1 = 1), and so forth. To interpret the effects of the predictors, the parameter estimates are exponentiated, resulting in a hazard ratio (due to the use of the cloglog link).

In the case of a large-*N* design without repeated measurements, the parameters of a discrete-time hazard model can be estimated using standard logistic regression software (after expanding the typical person-trial-oriented data set into a person-trial-bin-oriented data set; Allison, 2010). When there is clustering in the data, as in the case of a small-*N* design with repeated measurements, the parameters of a discrete-time hazard model can be estimated using population-averaged methods (e.g., Generalized Estimating Equations), Bayesian methods, or generalized linear mixed models (Allison, 2010). Examples of the latter can be found in Panis (2020), Panis et al. (2020), Panis and Schmidt (2016, 2020), and Wolkersdorfer et al. (2020). Finding the best random effects structure to generalize beyond the sample is an active area of research (Barr et al., 2013; Cunnings, 2012; Matuschek et al., 2017; Zuur & Ieno, 2016). Note that in case of a small-*N* design, EHA allows one to test if and how individual performance changes on multiple time scales (e.g., within-trial, across-trial, across-block).

When you treat time continuously, you can fit parametric models (e.g., a lognormal hazard model, an exponential hazard model, and so forth; Figure 1), semiparametric models such as the Cox regression model that ignores the shape of the hazard function and only tests the beta parameters, or piecewise exponential models (Allison, 2010). A piecewise exponential model is useful when (a) event times are measured precisely, (b) you want to estimate the shape of the hazard function, and (c) you do not want to impose a parametric model: Time is divided into intervals, and the hazard rate is assumed to be constant within each interval (i.e., exponentially distributed RTs within each interval).

The use of rather complex regression models to analyze hazard and conditional accuracy functions, and the employment of stepwise techniques to find the best model, harbor the danger of over- or underfitting the data, especially when the model is tested with the same data to which it was fitted. *P* values from such models have to be treated with the appropriate caution. Therefore, a third approach to obtain inferential statistics is to define different parameters of the descriptive functions (e.g., onset thresholds, divergence and convergence points, inflection points, and so forth) and to use robust techniques such as bootstrapping and jackknifing to compare and test their distributions (Ulrich & Miller, 2001; Wilcox, 2011).

We can shortly illustrate a very simple and immensely useful jackknifing procedure suggested by Ulrich and Miller (2001). Consider the data in Figure 4A (left panel), where we found that the hazard function for incongruent trials experiences a temporary drop in performance (Panis & Schmidt, 2016). If we know from previous experiments that such effects can take place in a certain time window, we can use that window as a region of interest (ROI). The jackknifing procedure now consists of extracting subsamples of the data, each of which contains the average curve for the incongruent trials within the ROI *except for one participant*. Each subsample excludes a different participant so that we have as many subsamples as participants (*N*). The advantage is that each subsample contains a relatively smooth curve that is based on *N* – 1 participants. It is therefore much easier to extract parameters of interest from each subsample curve than trying the same for the noisy data of single participants. For example, we can easily find the bottom of the dip in hazard in incongruent trials and extract its time (or amplitude, or both) for each subsample. Those *N* values can now be put into a table and used for standard ANOVA. Of course, the mean of the subsample curves will be identical to the mean of the individual participants’ curves, but the variance will be too small because each participant is included *N* – 1 times. Therefore, all *F* values have to be corrected by dividing them by a factor of (*N* – 1)^2^, and the *p* values have to be recalculated accordingly (for proofs, see Ulrich & Miller, 2001).

## Discussion

### The Theoretical and Statistical Advantages of EHA

Many experimental psychologists are still reluctant to embrace EHA and to stop using ANOVA when dealing with time-to-event data. In part, this is due to historical reasons. The computer metaphor of cognition—serial information processing via consecutive stages—was developed by Donders (1969) and became very popular from 1960 onward (Sternberg, 1969, 1984, 2013). During the past decades, however, various distributional methods have been advertised to move beyond the mean (Balota & Yap, 2011; Ridderinkhof, 2002; van Maanen et al., 2019; VanRullen, 2011).

Nevertheless, while many still assume that RTs reflect the cumulative duration of all time-consuming cognitive operations involved in a task (e.g., Liesefeld, 2018; Song & Nakayama, 2009), the results from various discrete-time event history and conditional accuracy analyses show that fast, medium, and slow RTs can actually index different sets of cognitive operations (Figures 4 and 6; cf. van Zoest et al., 2010). Statistically controlling for the passage of time on multiple time scales during data analysis is therefore *equally* important as experimental control during the design of an experiment, to understand human behavior in our experimental paradigms (Panis, 2020; Panis & Schmidt, 2016, 2020).

The distributional data in Figures 4 [Fig fig5-2041669520978673]to 6 are consistent with a dynamic systems approach to cognition according to which cognition involves sequential transitions between stable sensory, motor, and central states (Schöner et al., 2016). To understand the behavioral output of the brain, we must therefore measure quantities—*h*(t) and *ca*(t)—that track the motor states over time to study how long they last, how they are replaced by new states, and whether and when different manipulations affect them, to try to infer the spatial-temporal interplay between different cognitive component processes. Averaging these processes over time to look at mean RTs only sometimes preserves the crucial information in the time course of motor activity. More often than not, mean performance measures paint a picture that distorts, conceals, or even reverses the actual dynamical events. One example is the analysis in Figure 5B, which reveals a sequence of positive priming followed by a negative compatibility effect (Panis & Schmidt, 2016). An analysis in terms of mean error rate would necessarily miss at least one of these phases because the effect in mean error rate can only be positive or negative, but not both. It may even miss both phases if integration over time leads to an average that is too small to be significant.

Statistical reasons in favor of EHA include the ability to deal with right-censored observations and time-varying covariates and the fact that hazard provides exactly the kind of information we want to extract from RT data: the instantaneous likelihood of event occurrence given no previous events. We thus recommend to always first plot the *h*(t) and *ca*(t) functions of each individual (small-*N* design) or group of experimental units (large-*N* design) before making any further data-analytic or computational modeling decision. This practice would also inform the field about the various shapes the hazard function can take on in different contexts—a big unknown—and this will help in choosing which (combination of) parametric functions we might want to fit to the data, and in knowing how complex our computational models have to be to capture the behavioral dynamics observed empirically (Holden et al., 2009; Townsend & Ashby, 1983; Wickens, 1982).

Issues about bin size optimality play a secondary role at this moment in time in our view, because by working in discrete time—or using interval-censored data—we can make an informed trade-off between the availability of temporal information (smaller bins increase temporal resolution) with the feasibility to perform expensive data collection efforts (small bins can only be used with a large number of repeated measurements in case of a small-*N* design). In other words, the number and sizes of the time bins used for the analyses can be optimally adapted to each situation, depending on the duration of the data collection period, the rarity of event occurrence, the shape of the empirically observed hazard function, and whether one is using a large- or small-*N* design (Smith & Little, 2018).

As a standard method, EHA offers a unifying and principled approach to the analysis of time-to-event data that can be flexibly combined with other tools used by cognitive (neuro)scientists. For example, by transforming a sample of time-to-event data into time-series data—*h*(t) and *ca*(t) functions—one puts the analysis of behavior on the same footing with respect to time as physiological data such as EEG. Incorporating time-varying covariates (e.g., occipital EEG power in the alpha band) in hazard models of behavioral (or neural) event occurrence extends the set of current approaches to perform cognitive psychophysiology (Meyer et al., 1988). Also, combining EHA with transcranial magnetic stimulation (TMS) allows to read out the time-dispersed effect of a timed TMS pulse in the *h*(t) and *ca*(t) functions to answer the question: “When is area x necessary for task y?”

Finally, as explained by Kelso et al. (2013), it is crucial to first have a precise description of the macroscopic behavior of a system (here: *h*(t) and *ca*(t) functions) in order to know what to derive on the microscopic level. For example, fitting parametric functions or computational models to data without studying the shape of the *h*(t) and *ca*(t) functions can miss important features in the data (Panis et al., 2020; Panis & Schmidt, 2020). Due to the advantages of EHA, we recommend that it is used more often in future empirical and simulated RT studies. R code to calculate the descriptive statistics and the inferential statistics used by discrete-time EHA for a factorial within-subject design can be downloaded here: https://www.researchgate.net/publication/304069212_What_Is_Shaping_RT_and_Accuracy_Distributions_Active_and_Selective_Response_Inhibition_Causes_the_Negative_Compatibility_Effect.

### Discrete-Time EHA Versus Other Distributional Methods

#### Continuous-Time EHA

Discrete-time methods treat time-to-event data as interval-censored data while continuous-time methods use the exact event times. While learning the discrete-time methods first will ease the learning of the more complex continuous-time methods, they also have a lower temporal resolution. Thus, although statistical modeling of continuous time-to-event data requires specialized software to either fit parametric hazard models that are rather restrictive in the shapes they allow (e.g., a Weibull hazard model), or semiparametric hazard models that completely ignore the shape of the hazard function, their use might be warranted in particular circumstances. Allison (2010) provides a useful list of considerations when choosing between discrete- and continuous-time methods to perform an EHA. An overview of R functions for a continuous-time EHA can be found here: https://cran.r-project.org/web/views/Survival.html.

#### Quantile Plots and Classic Delta Plots

A quantile plot visualizes a set of quantiles (e.g., the nine deciles) as a function of quantile order. A classic delta plot for RT compares two conditions by subtracting corresponding quantiles and plots each of these (e.g., nine) differences (*y* axis) as a function of the average of both quantiles in question (*x* axis). This way we can easily examine in which range of RTs the effect in *F*(t) is large or small, positive or negative. However, if participants vary strongly in the identity of the time bin in which their fastest emitted responses occur, then quantiles will be very variable among participants, and averaging them will result in a blurring of effects that might otherwise be time-locked to the onset of a stimulus, for example—and effect sizes can also be attenuated. Therefore, we recommend simply plotting the difference in hazards or conditional accuracies for each bin (as in Panis, 2020, Panis & Schmidt, 2020).

Procedures such as Vincentizing (construction of average RT distributions from the average of their quantiles) that are assumed to normalize the RT distributions across participants (Ratcliff, 1979) have not been evaluated positively (Rouder & Speckman, 2004). Instead, we believe that if, for example, the range of RTs and the time course in hazard of an effect are different across participants, then this is theoretically interesting and requires a substantial explanation. Even if it is possible to somehow average those distributions, that does not mean that the underlying processes should be lumped together. Note that individual differences (e.g., in working memory capacity, the time required to stop a response, and so forth) can be taken into account by adding relevant predictors to the participant level of a multilevel hazard model, thus allowing for participant effects and cross-level interactions.

#### Possible Disadvantages of Discrete-Time EHA

There are also possible disadvantages of discrete-time EHA.

First, the person-trial-bin-oriented data set can become very large.

Second, one needs to explore a few bin sizes to find the optimal size for a particular data set. The optimal bin size will depend on the censoring time, the overall rarity of event occurrence, and the number of repeated measures or trials (small-*N* design) or the number of participants (large-*N* design). Note that the time bins do not have to be all of equal size (Panis, 2020).

Third, in hierarchical data from a small-*N* design, there are two sources of noise: within and between participants. For a distributional analysis, it is important to have enough repeated measures per participant and condition (preferably at least 100) to minimize the influence of within-participant noise. Between-participant variation is a different matter: It can be due to noise but also due to characteristic differences between individuals (e.g., in speed, capacity, or strategy). Again, high measurement precision in single participants and the incorporation of covariates at the participant level in a multilevel model is the only way to deal with this. *Power contours* can be used to estimate how many repeated measures are required to reach 80% power for a given sample size *N*, and vice versa (Baker et al., 2020; see their paper for a useful online tool).

In general, analyzing single participants should be regarded as a safeguard against interpreting spurious effects in the pooled data that are actually only generated by a minority of participants while at the same time refraining from overinterpreting the individual data patterns. Note that systematic effects will be visible for a majority of participants, while occurrences due to noise will not.

### Recommendations for Experimental Design of RT and Other Time-to-Event Data Studies

Two general recommendations can be made from the viewpoint of EHA when designing RT studies. First, always use the same fixed response deadline in each trial, for example, 500 ms for single-button detection and 800 ms for an easy two-button discrimination task. Because hazard analysis deals with right-censored observations, there is no need to wait for very slow responses that are considered meaningless and would be trimmed anyway. Also, using rather short and fixed response deadlines will lead to individual distributions that overlap in time, which is important for *h*(t) and *ca*(t) modeling (Panis & Schmidt, 2016). Furthermore, if you wait for a response in each trial and let the overt response end the trial, then you allow participants to have control over the trial (and experiment) duration, which can be avoided (or systematically controlled).

Second, try to design as many trials as possible per condition because then you can use small bins and still obtain stable *h*(t) and *ca*(t) estimates (i.e., use a small-*N* design; Smith & Little, 2018). Also, designing 100 trials per condition, for example, will not result in a large increase in experiment duration as the response deadline and thus trial duration can be kept short (see Panis & Schmidt, 2016). Note that many more trials are needed if you want to characterize the detailed shape of the right tail of a RT hazard distribution, especially in continuous time.

## Conclusions

RT and accuracy distributions are a rich source of information on the time course of cognitive processing. The changing effects of our experimental manipulations with increases in waiting time become strikingly clear when looking at response hazards and microlevel speed-accuracy trade-off functions. Indeed, working with hazard and conditional accuracy functions, you will discover a whole new layer of the data, and presumably the one where the processes live that actually interest you. An EHA of time-to-event data can strongly constrain the choice between cognitive models of the same psychological phenomenon. Due to the theoretical and statistical advantages of EHA, the fundamental simplicity of the method, and the availability of free software, there is currently no reason anymore to not start using EHA for time-to-event data.
